# Rapid Spread of *Tomato Yellow Leaf Curl Virus* in China Is Aided Differentially by Two Invasive Whiteflies

**DOI:** 10.1371/journal.pone.0034817

**Published:** 2012-04-13

**Authors:** Huipeng Pan, Dong Chu, Wenqian Yan, Qi Su, Baiming Liu, Shaoli Wang, Qingjun Wu, Wen Xie, Xiaoguo Jiao, Rumei Li, Nina Yang, Xin Yang, Baoyun Xu, Judith K. Brown, Xuguo Zhou, Youjun Zhang

**Affiliations:** 1 Department of Plant Protection, Institute of Vegetables and Flowers, Chinese Academy of Agricultural Sciences, Beijing, People's Republic of China; 2 High-tech Research Center, Shandong Academy of Agricultural Sciences, Jinan, People's Republic of China; 3 School of Plant Sciences, The University of Arizona, Tucson, Arizona, United States of America; 4 Department of Entomology, University of Kentucky, Lexington, Kentucky, United States of America; Max Planck Institute for Chemical Ecology, Germany

## Abstract

**Background:**

*Tomato yellow leaf curl virus* (TYLCV) was introduced into China in 2006, approximately 10 years after the introduction of an invasive whitefly, *Bemisia tabaci* (Genn.) B biotype. Even so the distribution and prevalence of TYLCV remained limited, and the economic damage was minimal. Following the introduction of Q biotype into China in 2003, the prevalence and spread of TYLCV started to accelerate. This has lead to the hypothesis that the two biotypes might not be equally competent vectors of TYLCV.

**Methodology/Principal Findings:**

The infection frequency of TYLCV in the field-collected *B. tabaci* populations was investigated, the acquisition and transmission capability of TYLCV by B and Q biotypes were compared under the laboratory conditions. Analysis of *B. tabaci* populations from 55 field sites revealed the existence of 12 B and 43 Q biotypes across 18 provinces in China. The acquisition and transmission experiments showed that both B and Q biotypes can acquire and transmit the virus, however, Q biotype demonstrated superior acquisition and transmission capability than its B counterparts. Specifically, Q biotype acquired significantly more viral DNA than the B biotype, and reached the maximum viral load in a substantially shorter period of time. Although TYLCV was shown to be transmitted horizontally by both biotypes, Q biotype exhibited significantly higher viral transmission frequency than B biotype. Vertical transmission result, on the other hand, indicated that TYLCV DNA can be detected in eggs and nymphs, but not in pupae and adults of the first generation progeny.

**Conclusions/Significance:**

These combined results suggested that the epidemiology of TYLCV was aided differentially by the two invasive whiteflies (B and Q biotypes) through horizontal but not vertical transmission of the virus. This is consistent with the concomitant eruption of TYLCV in tomato fields following the recent rapid invasion of Q biotype whitefly in China.

## Introduction

*Tomato yellow leaf curl virus* (TYLCV) is a single stranded DNA (ssDNA) plant virus in the genus *Begomovirus*, family *Geminiviridae*. Begomovirus genomes are either a monopartite (DNA-A) or bipartite (DNA-A and DNA-B) and require a single coat protein molecule for encapsidation into a twinned icosahedral or ‘geminiate’ particle [1], [2]. Begomoviruses are transmitted by the sweet potato whitefly, *Bemisia tabaci* (Gennadius) (Hemiptera: Aleyrodidae), in a circulative manner and are persistent in the whitefly vector [3]–[6]. TYLCV, originated in the Middle East-Mediterranean region [7], has been introduced into many other regions around the world making it among the most virulent and damaging begomoviruses in tomato crops. Symptoms of TYLCV infection are leaf curling, overall stunting, and yield loss of tomato plants ranging from 20–100% depending on the stage of plant growth at the time of infection. TYLCV recently has become a worldwide insect-borne plant disease in tomato, other vegetable crops, and ornamentals due to multiple introductions of the virus and the invasive *B. tabaci* B and Q biotypes that transmit it [6], [8].

In China, the presence of TYLCV has been documented in 6 provinces in the past 5 years. The exotic virus was first detected in symptomatic tomato plants in March 2006 in Shanghai, China [9]. Subsequent monitoring showed that TYLCV also had invaded Zhejiang Province during the autumn-winter cropping season of 2006 [10]. Since then it has moved toward northern part of the China to Jiangsu, Shandong, Beijing, and Hebei provinces where it has caused unprecedented economic losses, particularly in tomato crops [11]–[14].

The acquisition and transmission of TYLCV through their insect vectors has been a research focus for the past decade. Several lines of evidence have suggested that TYLCV can be transmitted both horizontally by sexual transmission and vertically via transovarial passage [15], [16]. These transmission routes may exert dramatic effects on virus epidemiology [17]. Ghanim and Czosnek (2000) demonstrated that horizontal transmission played a key role in transmitting TYLCV to tomato plants through infected whiteflies [18]. The bipartite begomoviruses *Squash leaf curl virus* (SLCV) and *Watermelon chlorotic stunt virus* (WmCSV) were transmitted horizontally among whiteflies with an efficacy similar to that of TYLCV [16]. In China, TYLCV and *Tomato yellow leaf curl China virus* (TYLCCNV) were shown to be horizontally transmitted by both B and Q biotypes, but transmission frequency was low [19].

On the other hand, TYLCV can be acquired by whiteflies independent of the infected plant source, i.e., the virus can be transmitted either horizontally or vertically [20]. Ghanim et al. (1998) demonstrated that TYLCV could be passed onto whitefly progeny, and the progeny of viruliferous insects can infect tomato plants [15]. Similar to TYLCV, a closely related *Tomato yellow leaf curl Sardinia virus* (TYLCSV) was found to be transmitted vertically to offspring [17]. Unlike TYLCV, however, the viruliferous progeny did not infect tomato plants [17].

The *B. tabaci* species complex is composed of closely-related sibling species. Each species is made up of a group of genetically divergent but morphologically indistinguishable haplotypes, that when well characterized are referred to as ‘biotypes’ [5], [6]. These biotypes differ in host range, virus transmission, insecticide resistance, and endosymbionts [21]–[24]. It has also been proposed that *B. tabaci* is better represented as a collection of discrete species [21], [25], [26], however, the absence of a tractable morphological and/or genetically based rationale for splitting groups of variants into separate species makes this suggestion taxonomically premature [5], [6]. Among this sibling species group are many haplotypes or variants, however, the B and Q biotypes have become prominent and are well known due to their invasive qualities, and a nearly worldwide distribution in agricultural and horticultural settings [25].

*B. tabaci* was first recorded in China in the late 1940 s [27]. However, the crop damages and economic losses caused by this phloem-feeding insect had not been severe until the introduction of *B. tabaci* B biotype in the mid-1990 s [28]. Since then, B biotype has quickly displaced the indigenous *B. tabaci* populations, rapidly invaded the entire country, and has led to serious yield losses in many crops [29]. In 2003, Q biotype was first detected in Yunnan Province of China as a new invasive whitefly [29] and just like B biotype, it has quickly adapted and established in many provinces of the mainland China [30], [31]. During the past few years, Q biotype has gradually displaced earlier well-established B biotype populations in the field and has become the dominant *B. tabaci* biotype in most parts of China [32]–[35].

In many regions of the world, outbreaks caused by whitefly-transmitted viruses have concurred with invasions of *B. tabaci* B and/or Q biotype [2], [5], [8], [36], [37]. In China, TYLCV has not been detected until the *B. tabaci* Q biotype became established, even though B biotype, an important vector of TYLCV elsewhere, has been in China since the mid-1990's [28], [31]–[35]. We hypothesize that the introduction and spread of TYLCV in China is closely related with the establishment of *B. tabaci* Q biotype. To test this hypothesis, we 1) surveyed the frequency of TYLCV in the field-collected *B. tabaci* B and Q populations; 2) compared the virus acquisition capability of the two biotypes; and 3) investigated the mode of viral transmission (horizontal and vertical) under controlled laboratory conditions.

## Materials and Methods

### *Bemisia tabaci* field collections and biotypes determination

**Figure 1:**
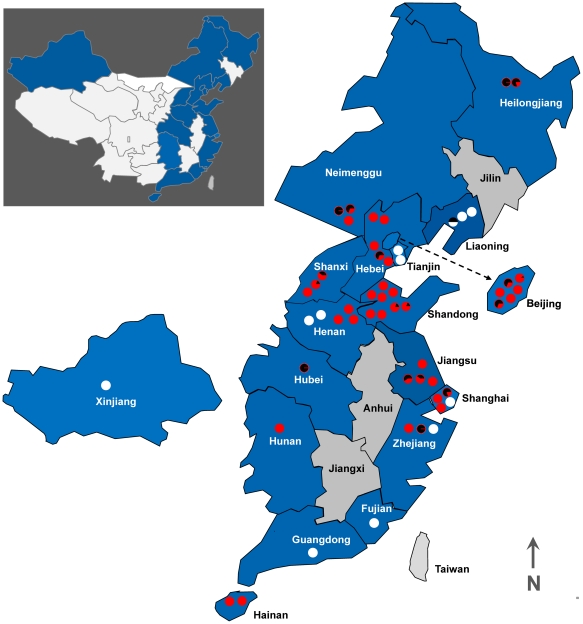
TYLCV distribution pattern among *B. tabaci* B and Q biotypes in China. The survey was carried out in the field season of 2009. The white and red dots represent *B. tabaci* B and the Q biotype, respectively. The black portion of the dots denotes TYLCV-infected *B. tabaci* population and its respective infection rate. Inset: Outline map of China showing all the provinces. Surveyed provinces are highlighted in a blue shade.

*B. tabaci* populations were sampled from 55 field sites throughout 18 provinces of China during the 2009 crop season (Figure 1, Table S1). At each site, three subsamples of whiteflies were collected, with an approximately 500–1000 m distance in between each subsample. The whiteflies from three subsamples were combined into one collection per site. At least 100 whiteflies per collection site were preserved in 95% ethanol and stored at −20°C. Genomic DNA was extracted from each of 50 individual whiteflies per collection site according to De Barro and Driver (1997) and Frohlich et al. (1999) [38], [39], and stored at −20°C until analysis. The whitefly biotypes were determined by the CAPS (cleavage amplified polymorphic sequence) of *mtCOI* (mitochondrial cytochrome oxidase I) with the restriction endonucleases *Vsp*I [32]. The presence of TYLCV was assessed for 50 individuals from each collection site.

### *Bemisia tabaci* laboratory population

The B biotype population was originally collected from a field-infested cabbage, *Brassica oleracea* L. cv. Jingfeng 1, in Beijing, China in 2004 [40], whereas, the Q biotype was collected from the poinsettia, *Euphorbia pulcherrima* Wild. ex Klotz., in Beijing, China in 2009. The B and Q biotype populations have been provisioned with tomato plants, *Solanum lycopersicum* Mill. cv. Zhongza 9, and maintained in isolated whitefly-proof screen cages in a greenhouse under natural lighting and controlled temperature (26±2°C) for 6 generations before subjected to this experiment. The purity of these populations was monitored by sampling 15 adults per generation using a molecular diagnostic technique, CAPS (cleavage amplified polymorphic sequence), and a molecular marker, mitochondrial cytochrome oxidase I genes (*mtCOI*) [32].

### Acquisition of viruliferous and non-viruliferous *B. tabaci*

TYLCV-infected tomato plants were obtained by *Agrobacterium tumefaciens*- mediated inoculation using a cloned TYLCV genome (GenBank accession ID: AM282874), which was originally isolated from tomato plants in Shanghai, China [9]. Inoculation was carried out at the 3 true-leaf stage. Viral infection of test plants was confirmed by the development of characteristic leaf curl symptoms and was further validated by the molecular analysis, as described below. Viruliferous *B. tabaci* were obtained by caging adults (2 days post-emergence) with a TYLCV-infected tomato plant for a 72 h acquisition access period (AAP) [41]. Accordingly, non-viruliferous adults were collected from virus-free tomato plants two days post-inoculation and caged onto leaves of uninfected tomato plants for a 72 h AAP.

### Acquisition of TYLCV by *B. tabaci* B and Q biotypes

Approximately 350 newly emerged (0–8 h post-emergence) adults were placed on TYLCV-infected tomato plants. Thirty adults (females and males approximately in equal numbers) were collected randomly following a 6, 12, 24, 48, 72 h acquisition access period (AAP) on TYLCV-infected tomato plants, and stored at −80°C. DNA from the batches of 30 viruliferous whiteflies was extracted using Universal Genomic DNA Extraction Kit Ver.3.0, Cod: DV811A (TaKaRa Biotechnology, Dalian Co., Ltd). The design of TYLCV qRT-PCR primer set, TY-F (GTCTACACGCTTACGCC) and TY-R (GCAATCTTCGTCACCC) was based on a consensus of eight different TYLCV isolates. qRT-PCR reaction was carried out in a 96-well optical plate in the Applied Biosystems 7500 real time PCR instrument. The cycling condition was: 5 min activation at 94°C, 40 cycles of 30 s at 94°C, 30 s at 60°C, and 1 min at 72°C. For each sample, three replicates were amplified in each of the three biologically independent experiments. The relative gene expression of TYLCV was calculated using the 2^−ΔCt^ method [42]. *β-actin* from *B. tabaci* was used as a reference gene to normalize the gene expression level.

### Modes of viral transmission

#### Horizontal transmission of TYLCV via mating

Two sets of experiments were carried out for the *B. tabaci* biotype-virus combinations (B and Q biotypes with TYLCV, respectively). Each set of experiments consisted of four treatments (T1–T4) with 25 replications for each treatment. Specifically, these treatments included, one viruliferous female paired with one non-viruliferous male (T1), one non-viruliferous female paired with one viruliferous male (T2), two viruliferous females paired with two non-viruliferous males (T3), and two non-viruliferous females paired with two viruliferous males (T4). Whitefly adults for each replicate and treatment were enclosed in a clip-cage and placed on the lower surface of a cotton leaf. The adults were left on the leaf to feed and mate for 72 h at 26±1°C, 50% relative humidity and a photoperiod of 14: 10 (L : D). Then, all whitefly adults were collected and stored at −20°C for the subsequent detection of viral DNA.

**Figure 2:**
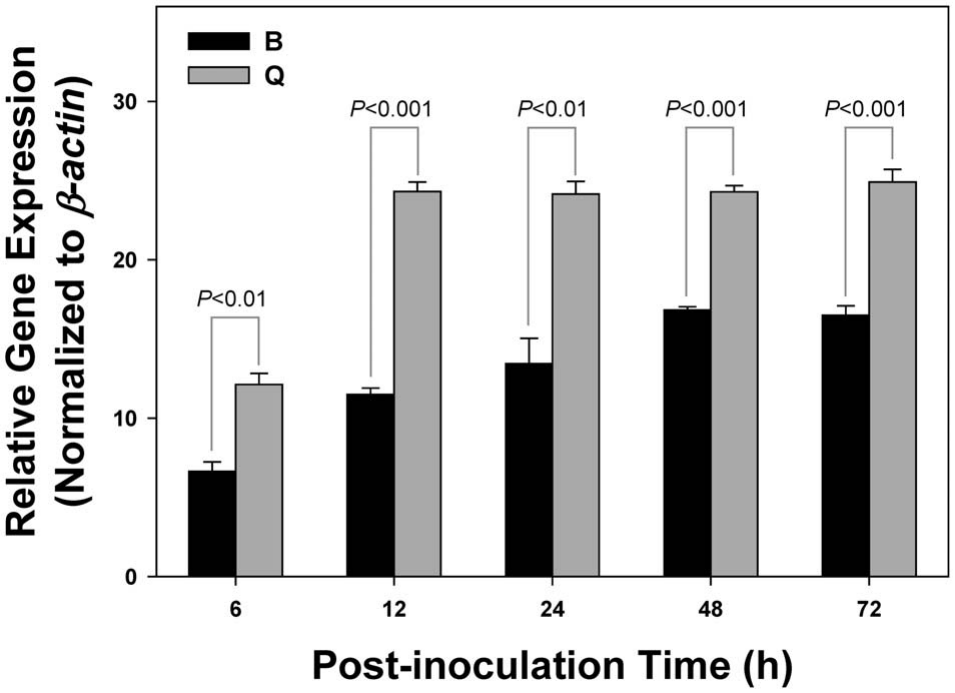
Acquisition of TYLCV by *B. tabaci* B and Q biotypes. Relative gene expressions of TYLCV in *B. tabaci* B and Q biotypes were measured using a qET-PCR after whiteflies fed on the TYLCV-infected tomato plants for 6, 12, 24, 48, and 72 h. Statistical differences are denoted by the *p*-value using a Chi-square test.

#### Vertical transmission of TYLCV via inheritance

Similar to the horizontal transmission design, two sets of experiments were performed. For each set, four developmental stages (egg, crawler, pupa, and adult) of the progeny of viruliferous adults were tested for the presence of TYLCV DNA. Specifically, one non-viruliferous adult male and one viruliferous adult female were transferred to the lower surface of a cotton plant leaf and enclosed in a clip-cage. The adults were left to feed, mate, and oviposit for 5 days at 26±1°C, 50% relative humidity, and a photoperiod of 14: 10 (L: D). Then the *B. tabaci* adults were removed and the eggs were left on the leaf to develop. Forty replicates were carried out for the whitefly biotype-TYLCV combinations. The replicates in each of the two sets were divided evenly into two groups. In accordance with the developmental periods of whitefly [15], sampling was timed to collect the progeny at different stages. Four days after removing adults, progeny of one group were sampled. Five eggs and five crawlers from each replicate were collected using disposable sterilized needles (one needle for each individual) and stored at −20°C for detection of viral DNA. Twenty days after removing adults, progeny of the second group were sampled. Five pupae and five adults from each replicate were collected using disposable sterilized needles (one needle for each individual) and stored at −20°C for detection of viral DNA.

**Figure 3:**
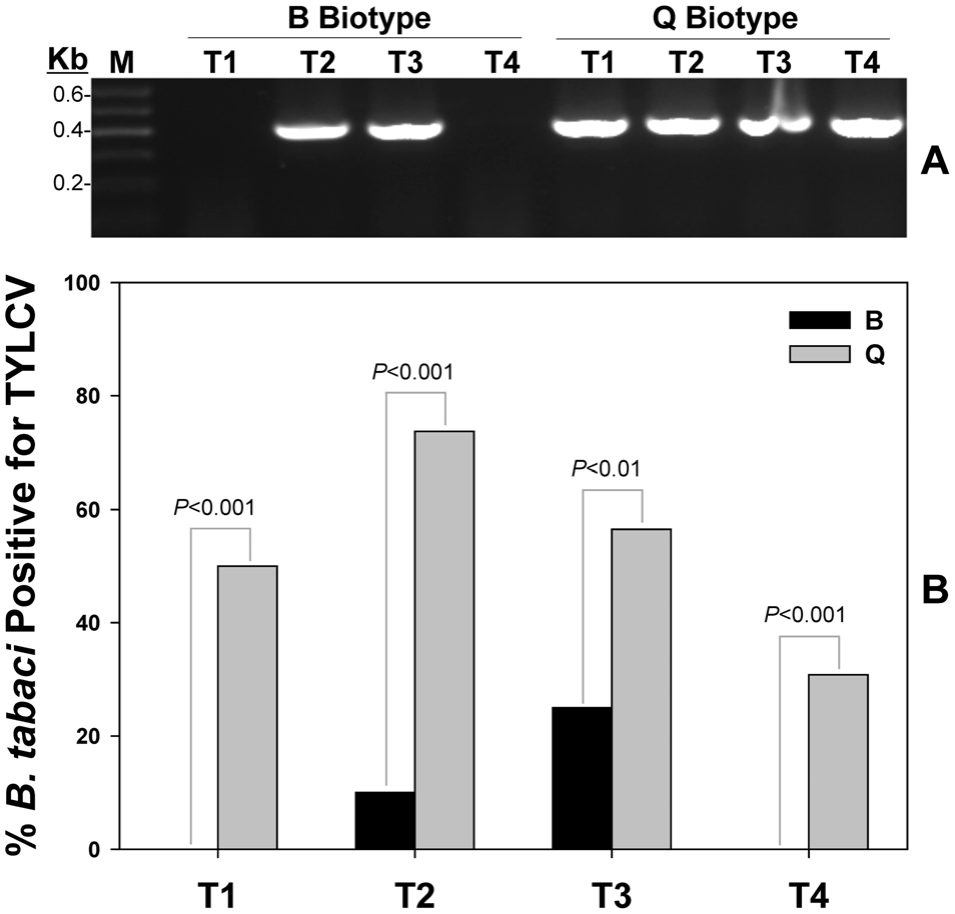
Horizontal transmission of TYLCV by *B. tabaci* B and Q biotypes. Transmission of TYLCV from viruliferous to non-viruliferous *B. tabaci* of the opposite sex was evaluated under four different treatments: T1 (1F*×1M^0^), T2 (1F^0^×1M*), T3 (2F*×2M^0^), and T4 (2F^0^×2M*). F*, viruliferous female; F^0^, initially non-viruliferous female; M*, viruliferous male; M^0^, initially non-viruliferous male. A total of 25 replications were included in each of these treatments. Detection of viral transmission was confirmed by the presence of a 400 bp amplicon of TYLCV DNA on an agarose gel (**A**). M, DNA size marker (Marker I, GIBCO-BRL). The percentage of whiteflies tested positive for TYLCY was summarized in **B**. Statistical differences are denoted by the *p*-value using a Chi-square test.

### Viral DNA detection in *B. tabaci* and tomato plant, respectively

Genomic DNA was extracted from *B. tabaci* pupa and adult, respectively, according to De Barro and Driver (1997) and Frohlich et al. (1999) [38], [39], A different procedure, however, was used for the egg and the crawler stage. DNA was extracted by crushing a single egg or crawler with a sterile needle once placed into 15 µl of 0.5×TE buffer in a 0.5 ml tubes. The suspensions were homogenized by a 30 min treatment in an ultrasonic bath, and then boiled for 5 min. Following brief centrifugation, extracts were used immediately subjected to the Whole Genome Amplification (WGA). The DNA extracts were first analyzed for the presence of *B. tabaci* DNA. Before PCR, all preparations of insect DNA were subjected to the WGA, using Universal WGA Kit (Tiandz, Inc, Beijing, China) according to the manufacturer's protocol. The WGA products were used as templates for the subsequent PCR. The quality of the whitefly DNA preparation was assessed by PCR-amplification of a 372 bp fragment of *B. tabaci* ketose reductase (KR) gene [17]. Two primers, TYLCV-61 and TYLCV-473 that amplified the AV2 gene, were used to detect the presence of TYLCV [16].

The cotton plants used to feed *B. tabaci* in this study are known to be immune to TYLCV. A negative control was included to eliminate the possibility that non-viruliferous *B. tabaci* might acquire virus through feeding on the cotton plants previously infested with viruliferous whiteflies. To explore this possibility, 8 viruliferous males of B-TYLCV and Q-TYLCV combinations, respectively, were transferred to the lower surface of a cotton leaf and allowed to feed for 72 h in a clip-cage. A total of 8 replicates were conducted for each combination. Then total DNA from the leaves had been fed by viruliferous adults were extracted using the method of Xie et al. (2002) [43], and viral DNA was detected as described above.

### Data analysis

The different viral acquisition capability between B and Q biotypes at various AAP was analyzed using one-way analysis of variance (ANOVA). The frequency of TYLCV infection in *B. tabaci* B and Q biotype individuals and populations, the viral transmission between each biotype and TYLCV, and the virus-*B. tabaci* life stage combinations of the biotype, were analyzed by the Chi-square analysis using SPSS (SPSS for Windows, Rel. 17.0.0. 2009. Chicago: SPSS Inc.).

## Results

### The distribution of TYLCV among *B. tabaci* B and Q biotypes in China

PCR amplification of the AV2 gene of TYLCV resulted in the detection of a 400 bp amplicon among18 out of 55 collection sites across11 provinces, including Heilongjiang, Liaoning, Neimenggu, Hebei, Beijing, Shandong, Shanxi, Jiangsu, Zhejiang, Shanghai, and Hubei. TYLCV was not detected in the field-collected *B. tabaci* in the other 37 sites from 7 provinces, including Tianjin, Henan, Hainan, Hunan, Xinjiang, Fujian, and Guangdong (Figure 1, Table S1). Among these collection sites, 43 populations from 14 provinces were determined to be the Q biotype, while 12 populations from 8 provinces were identified as the B biotype (Figure 1, Table S1). Among the Q biotype populations, 17 (39.5%) were infected with TYLCV, while only 1 B biotype populations (8.3%) was infected. Similarly, the percentage of TYLCV-infected individuals was significantly lower among the B biotype (4.2%) than the Q biotype (24.4%).

**Figure 4:**
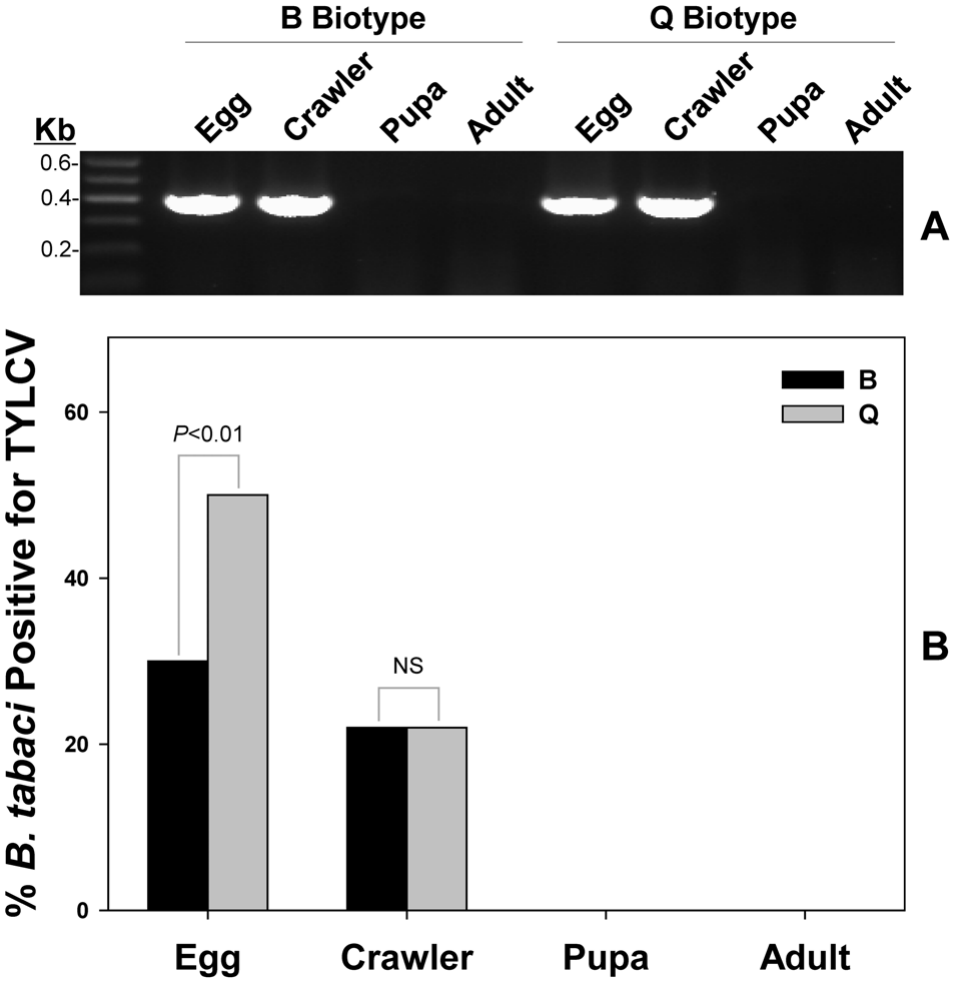
Vertical transmission of TYLCV by *B. tabaci* B and Q biotypes. A total of four developmental stages (egg, crawler, pupa, and adult) from the first generation progeny of viruliferous adults were tested for the presence of TYLCV DNA. Detection of viral transmission was confirmed by the presence of a 400 bp amplicon of TYLCV DNA on an agarose gel (**A**). M, DNA size marker (Marker I, GIBCO-BRL). The percentage of whiteflies tested positive for TYLCY was summarized in **B**. Statistical differences are denoted by the *p*-value using a Chi-square test. NS stands for not significant.

### Acquisition of TYLCV by *B. tabaci* B and Q biotypes

In this study, the qRT-PCR method was adopted to compare the virus acquisition capability between B and Q biotypes. The relative viral gene expression level of Q biotype was significant higher than that of the B biotype in each of the five AAP (Figure 2). In addition, *B. tabaci* B and Q biotypes needed 48 and 12 h AAP, respectively, to reach their respective maximum viral loads (Figure 2).

### Horizontal transmission of TYLCV

Two sets of experiments were carried out for the biotypes-virus combinations: B biotype-TYLCV and Q biotype-TYLCV. Results from non-viruliferous adults indicated that all four treatments involving the Q biotype-TYLCV combination were tested positive for the horizontal transmission, whereas only T2 and T3 treatments involving the B biotype-TYLCV combination were tested positive (Figure 3). Specifically, for the Q biotype-TYLCV combination, viruliferous females passed on the virus to 50% of the males in treatment (T1). Viruliferous males passed on the virus to 73.7% of the females in treatment (T2). Viruliferous females passed on the virus to 56.5% of the males in treatment (T3). Viruliferous males passed on the virus to 30.8% of the females in treatment (T4). For the B biotype-TYLCV combinations, viruliferous females failed to pass on the virus to the males in treatment (T1). Viruliferous males passed on the virus to 10.0% of the females in treatment (T2). Viruliferous females passed on the virus to 25.0% of the males in treatment (T3). Viruliferous males failed to pass on the virus to the females in treatment (T4). For each treatment, the percentage of TYLCV transmission by the Q biotype was substantially higher than the B biotype, and the overall percentage of TYLCV transmission by the Q biotype was 52.2%, compared to 10.5% for the B biotype, suggesting that the particular whitefly biotype had a significant effect on the frequency of horizontal transmission of TYLCV.

### Vertical transmission of TYLCV

Both B and Q biotypes were found to transmit TYLCV vertically, in which TYLCV could be detected in the eggs and crawlers with infection rates of 50.0 and 22.0%, respectively, for the Q biotype individuals, and 30.0 and 22.0%, respectively, for the B biotype individuals. However, virus was not detected in the pupa or adult progeny from viruliferous B or Q whiteflies (Figure 4). Interestingly, an age-dependent pattern of transmission frequency was observed in the immature stages in which transmission frequency was higher at the egg stage, and decreased following the subsequent development of the whitefly offspring.

**Table 1:** Vertical transmission of TYLCV in *Bemisia tabaci*.

Virus^a^	Biotype	Life Stage^b^			Reference
		Egg	Nymph	Adult	
TYLCV	B	81% (46/57)	37% (26/58)	57% (46/81)	[15]
TYLCV	B	0% (0/100)	0% (0/100)	0% (0/125)	[17]
TYLCSV	B	9% (10/110)	29% (32/110)	2% (5/250)	[17]
TYLCV	B	28% (11/40)	8% (6/80)	0% (0/40)	[19]
TYLCCNV	B	19% (14/75)	11% (17/150)	0% (0/75)	[19]
TYLCV	B	30% (30/100)	11% (22/200)^c^	0% (0/100)	This study
TYLCV	Q	17% (13/75)	15% (22/150)	3% (2/75)	[19]
TYLCCNV	Q	1% (1/75)	1% (2/150)	0% (0/75)	[19]
TYLCV	Q	50% (50/100)	11% (22/200)^c^	0% (0/100)	This study

a *Tomato yellow leaf curl virus* (TYLCV), *Tomato yellow leaf curl Sardinia virus* (TYLCSV), and *Tomato yellow leaf curl China virus* (TYLCCNV).

b Infection percentage for each life stage is reported as % (infected individuals/tested individuals).

c The nymphal stage of *Bemisia tabaci* can be divided into four instars. The first instar, commonly known as the crawler, and the fourth instar, called the “pupa", were the whitefly nymphs used in this experiment. Data presented here (11%) was the percent average of crawler (22%) and pupa (0%) (see Fig. 4B).

## Discussion

### Concurrent spread of the epidemic TYLCV and the invasive *B. tabaci* Q biotype

The previous reports showed that TYLCV has been found only in 6 provinces during the past 5 years in China [9]–[14]. In the present study, TYLCV has been identified across 11 provinces in China. For the first time, TYLCV has moved into Heilongjiang, Liaoning, Shanxi, Neimenggu, and Hubei provinces. Based on the historical records and current results, it can be concluded that TYLCV has been spread gradually from south to north and from coastal areas to inland during the past 5 years. The concurrent invasion of *B. tabaci* Q biotype [31]–[35] and frequent outbreaks of TYLCV in tomato crops suggested that *B. tabaci* Q biotype might be a major biotic factor facilitates the rapid spread of this virus in China. This observation is consistent with a previous report [44], in which a Q biotype collected from southern Spain transmitted both TYLCV isolates (TYLCV-Sr or TYLCV-Is) more efficiently than the B biotype. The Q biotype infected a significantly higher percentage of the test plants in their controlled greenhouse study, suggesting that Q might be a more competent vector of the virus [44].

The intimate relationship between outbreaks of TYLCV and rapid spread of *B. tabaci* Q biotype has been carefully documented in the Shandong Province, China. Field surveys dated that *B. tabaci* Q biotype was introduced into the Shandong Province in 2005 when B was the dominant whitefly biotype in the area. Q started to displace B in 2007 and became the dominant *B. tabaci* biotype by 2009 [32], [33]. As for tomato yellow leaf curl disease caused by TYLCV, it occurred only sporadically in Heze and Zibo, Shandong Province in 2007, then it started to quickly move into adjacent regions in 2008, including Weifang, Liaocheng, Jining, Qingdao, and Zaozhuang, Shandong Province. Eventually, it became epidemic across the entire Shandong Province and caused severe yield losses in tomato production in 2009 [12]. The concurrent movement of TYLCV and *B. tabaci* Q biotype in Shandong Province clearly supports our hypothesis that Q might be a causative agent facilitating the rapid spread of TYLCV in China.

### Viral transmission capability of *B. tabaci* B and Q biotypes

Acquisition of TYLCV by *B. tabaci* B and Q biotypes exhibits substantial differences. Q biotype not only showed significantly higher viral gene expression level at all 5 acquisition access periods, it also reached the maximum viral load 4 times faster than its B counterparts (12 h in Q and 48 h in B). In addition, the observed infection frequency of TYLCV among *B. tabaci* field populations and the mode of transmission study provide additional empirical evidences to suggest a stronger association between TYLCV-Q than TYLCV-B biotype. The infection rate of TYLCV among Q field populations was significantly higher than B. Horizontal transmission appeared to be instrumental in increasing the number of whiteflies capable of transmitting virus to tomato plants for a given *B. tabaci* population [18]. In this study, Q demonstrated superior horizontal transmission capability (52.2% infection frequency) in comparison to its B counterparts (10.5% infection frequency). On the other hand, TYLCV can also be transmitted between whiteflies in a sex-dependent manner [18]. TYLCV was transmitted from viruliferous males to non-viruliferous females and from viruliferous females to non-viruliferous males. Transmission of TYLCV in a gender-dependent manner was shared with both B and Q biotypes, suggesting that this is a biologically conserved trait among whiteflies [16]. Our study showed that both B and Q biotypes were able to transmit TYLCV vertically to eggs and crawlers, but not to pupa or adult of the first generation progeny. Overall, transmission of viral DNA through the vector egg is relatively common; however, vertical transmission of the infectious agent through generation appears to be, at best, a rare event, reported only once [15, Table 1]. One possible explanation is that virus activates *B. tabaci* immune responses, including autophagy and antimicrobial peptide production, which could lead to the gradual decrease of viral titer within the viruliferous whitefly [45]. Furthermore, it appears that vertical transmission of TYLCV by *B. tabaci* has little or no bearing on the epidemiology of the TYLCD, at least, in the field. It is worth noting that some of the discrepancies observed between our results and previous studies [16,18,19, Table 1] could be due to environmental variations, the particular virus isolates examined, and/or the specific *B. tabaci* haplotypes used in the experiments.

Multiple factors contribute to the viral transmission in whiteflies, *B. tabaci* B and Q biotypes. It has recently been demonstrated that a 63-kDa GroEL homologue produced by the endosymbiotic bacteria *Hamiltonella* is essential for the circulative transmission of the virus in *B. tabaci*. Interactions between GroEL protein and TYLCV particles ensure the safe circulation of the virus in insect hemolymph [46]–[48]. In Israel, *Hamiltonella* has only been detected in B biotype, and B can efficiently transmit the virus. In contrast, Q does not harbor *Hamiltonella*, and it can barely transmit TYLCV. Based on these observations, a causal link between the transmission efficiency of TYLCV and the presence of *Hamiltonella* has been established [46]–[48]. In China, however, the role of *Hamiltonella* played in the viral transmission is unclear. A nation-wide survey in China showed that infection frequencies of *Hamiltonella* in 17 B and 44 Q field populations were equally high (46.7% and 46.5%, respectively) [24]. Moreover, the B and Q lab populations used in this study have a much higher infection rate of *Hamiltonella* than in the field (nearly 100% for both biotypes; HPP, unpublished data). Although both B and Q biotypes in China can transmit TYLCV, Q demonstrates superior acquisition and transmission capability than its B counterparts. These results suggest that factors other than *Hamiltonella* likely play a substantial role in mediating the acquisition and transmission ability of TYLCV between the two biotypes. Studies on the interactions between *B. tabaci* endosymbionts and TYLCV transmission are currently under investigation.

Previous studies have also shown that plant viruses can manipulate the biology of *B. tabaci* [41], [44], [49], [50]. In a parallel study, TYLCV-infected tomato plants improve the performance of Q biotype, whereas it exerts negative effects on the B biotype in regard to the fecundity, survival rate, development time, and female body size (HPP, unpublished data). Current study also suggests that the introduction of TYLCV, coupled with unfavorable environmental conditions in some locations, may influence the distribution profiles of *B. tabaci* B and Q biotypes in China.

### Summary

The combined field and laboratory observations demonstrate the potential for the differential spread of TYLCV by *B. tabaci* B and Q biotypes, which are both invasive and recently introduced *B. tabaci* vectors in China. These results in conjunction with previous studies suggest that the prevalence of *B. tabaci* Q biotype is linked to a greater disease incidence compared to the B biotype in China.

## Supporting Information

Table S1 **Collection information for** ***Bemisia tabaci*** **field populations.**
(PDF)
