# Supplementary material for: Rapid Spread of Tomato Yellow Leaf Curl Virus in China Is Aided Differentially by Two Invasive Whiteflies
Source: PLoS One. 2012 Apr 13;7(4):e34817. doi: 10.1371/journal.pone.0034817 (PMC3325912; doi:10.1371/journal.pone.0034817)
Supplement: Table S1 — Collection information for Bemisia tabaci field populations. (PDF) [file pone.0034817.s001.pdf]

Table S1. Collection information for *Bemisia tabaci* field populations

Population Number	Geographic Coordinates	Collection Site	Host Plant	Collection Month	Biotype
1	39° 18' N, 116° 58' E	Xiqing, Tianjin	Cotton	July	B
2	39° 9' N, 117° 24' E	Xiqing, Tianjin	Cucumber	September	B
3	34° 37' N, 112° 27' E	Luoyang, Henan	Cotton	August	B
4	34° 33' N, 112° 28' E	Luoyang, Henan	Cucumber	August	B
5	31° 13' N, 121° 19' E	Minhang, Shanghai	Cabbage	August	B
6	43° 48' N, 87° 34' E	Urumchi, Xinjiang	Cotton	September	B
7	26° 6' N, 119° 18' E	Fuzhou, Fujian	Sweet potato	September	B
8	30° 18' N, 120° 11' E	Hangzhou, Zhejiang	Tomato	September	B
9	23° 8' N, 113° 20' E	Guangzhou, Guangdong	Poinsettia	September	B
10	41° 52' N, 123° 24' E	Shenyang, Liaoning	Cucumber	September	B
11	41° 48' N, 123° 25' E	Shenyang, Liaoning	Tomato	September	B
12	41° 47' N, 123° 24' E	Shenyang, Liaoning	Beans	September	B
13	39° 51' N, 116° 19' E	Haidian, Beijing	Capsicum	July	Q
14	39° 56' N, 116° 20' E	Haidian, Beijing	Capsicum	July	Q
15	39° 57' N, 116° 16' E	Haidian, Beijing	Pimiento	July	Q

16	40° 13' N, 116° 13' E	Changping, Beijing	Cucumber	July	Q
17	39° 58' N, 116° 17' E	Haidian, Beijing	Tomato	July	Q
18	39° 53' N, 116° 18' E	Haidian, Beijing	Eggplant	July	Q
19	32° 23' N, 119° 23' E	Yangzhou, Jiangsu	Capsicum	July	Q
20	32° 21' N, 119° 24' E	Yangzhou, Jiangsu	Gerbera	July	Q
21	31° 34' N, 120° 18' E	Wuxi, Jiangsu	Cucumber	July	Q
22	20° 1' N, 110° 19' E	Haikou, Hainan	Eggplant	July	Q
23	20° 0' N, 110° 18' E	Haikou, Hainan	Towel Gourd	July	Q
24	32° 52' N, 120° 19' E	Dongtai, Jiangsu	Eggplant	July	Q
25	39° 32' N, 116° 41' E	Langfang, Hebei	Capsicum	July	Q
26	39° 33' N, 116° 42' E	Langfang, Hebei	Eggplant	July	Q
27	39° 27' N, 116° 38' E	Langfang, Hebei	Cucumber	July	Q
28	40° 49' N, 114° 52' E	Zhangjiakou, Hebei	Cucumber	September	Q
29	40° 48' N, 114° 55' E	Zhangjiakou, Hebei	Eggplant	September	Q
30	35° 12' N, 113° 2' E	Yucheng, Shanxi	Cucumber	August	Q
31	35° 32' N, 111° 11' E	Yucheng, Shanxi	Eggplant	August	Q
32	34° 16' N, 113° 24' E	Yucheng, Shanxi	Cotton	August	Q

33	34° 44' N, 113° 36' E	Zhengzhou, Henan	Cucumber	August	Q
34	34° 24' N, 113°19' E	Zhengzhou, Henan	Cotton	August	Q
35	34° 46' N, 113° 26' E	Zhengzhou, Henan	Eggplant	August	Q
36	35° 15' N, 115° 27' E	Heze, Shandong	Cotton	August	Q
37	35° 24' N, 115° 18' E	Heze, Shandong	Eggplant	August	Q
38	37° 26' N, 116° 21' E	Dezhou, Shandong	Eggplant	August	Q
39	36° 26' N, 116° 0' E	Liaocheng, Shandong	Japanese hop	August	Q
40	37° 15' N, 116° 41' E	Dezhou, Shandong	Cotton	August	Q
41	37° 36' N, 116° 43' E	Dezhou, Shandong	Japanese hop	August	Q
42	36° 39' N, 116° 59' E	Jinan, Shandong	Japanese hop	August	Q
43	36° 37' N, 116° 55' E	Jinan, Shandong	Cotton	August	Q
44	31° 12' N, 121° 25' E	Minhang, Shanghai	Tomato	August	Q
45	31° 13' N, 121° 29' E	Minhang, Shanghai	Eggplant	August	Q
46	31° 10' N, 121° 30' E	Minhang, Shanghai	Cucumber	August	Q
47	28° 11' N, 113° 1' E	Changsha, Hunan	Cotton	August	Q
48	45° 47' N, 126° 32' E	Haerbin, Heilongjiang	Cucumber	August	Q
49	45° 50' N, 126° 33' E	Haerbin, Heilongjiang	Tomato	August	Q

50	40° 50' N, 111° 45' E	Huhehaote, Neimeng	Cucumber	September	Q
51	40° 50' N, 111° 49' E	Huhehaote, Neimeng	Squash	September	Q
52	40° 49' N, 111° 42' E	Huhehaote, Neimeng	Bean	September	Q
53	30° 10' N, 120° 11' E	Hangzhou, Zhejiang	Eggplant	September	Q
54	30° 12' N, 120° 15' E	Hangzhou, Zhejiang	Tomato	September	Q
55	30° 35' N, 114° 16' E	Wuhan, Hubei	Cotton	September	Q
